# Stereotactic body radiotherapy delivered with IMRT for oligometastatic regional lymph node metastases in hepatocellular carcinoma: a single-institutional study

**DOI:** 10.1093/jrr/rraa067

**Published:** 2020-08-26

**Authors:** Munetaka Matoba, Hirokazu Tsuchiya, Tamaki Kondo, Kiyotaka Ota

**Affiliations:** Department of Radiology, Kanazawa Medical University; Department of Radiology, Kanazawa Medical University; Department of Radiology, Kanazawa Medical University; Department of Radiology, Kanazawa Medical University

**Keywords:** lymph node, metastasis, hepatocellular carcinoma, stereotactic body radiotherapy, intensity modulated radiotherapy

## Abstract

The optimal treatment to lymph node metastases in hepatocellular carcinoma (HCC) has not been established, yet. Our aim was to evaluate the local control, the survival benefit and the toxicity of stereotactic body radiotherapy (SBRT) delivered with intensity-modulated radiotherapy (IMRT) to oligometastatic regional lymph node in HCC patients. We retrospectively analyzed 15 patients with HCC treated with SBRT delivered using IMRT to 24 regional lymph node metastases. Dose prescriptions were set to 45 Gy in 6 fractions of 7.5 Gy for solitary lesions and 49.5 Gy in 9 fractions of 5.5 Gy for multiple lesions. For the planning target volume, the plan was optimized aiming for a *V*_95%_ > 90%. The study endpoints were freedom from local progression (FFLP), progression-free survival (PFS), overall survival (OS) and toxicity. The median follow-up was 18.1 months. The 1-year and 2-year FFLP rates were 100 and 90 ± 9.5%, respectively. The 1-year PFS rate was 46.7 ± 12.9%, and the 1-year and 2-year OS rates were 73.3 ± 11.4 and 28.6 ± 12.7%, respectively. Only one patient had a duodenal ulcer and three patients had liver enzyme elevation in sub-acute toxicity, however there was no grade ≥ 3 toxicity. In conclusion, SBRT delivered with IMRT to lymph node metastases can offer excellent local control with minimal toxicity, and SBRT may improve HCC patients’ survival more than conventional radiotherapy.

## INTRODUCTION

Abdominal lymph node metastases from hepatocellular carcinoma (HCC) are generally uncommon, but the reported incidence of lymph node metastases in HCC patients is 25–42% from autopsy studies [1]. The advances of diagnostic imaging techniques and nonsurgical treatments for HCC, such as transcatheter arterial chemoembolization (TACE), percutaneous ethanol injection, and radiofrequency ablation (RFA) have provided long-term control of HCC and survival benefit [2,3]. Consequently, the incidence of abdominal lymph node metastases that become clinically evident is likely to increase [4].

The prognosis for HCC patients with lymph node metastases is poor, with a median survival of < 4 months without treatment [5]. Metastatic lymph node involvement often introduces obstructions of the biliary system, the gastrointestinal tract, and/or the inferior vena cava, resulting in poor quality of life due to jaundice, abdominal pain, ileus and lower extremity edema [6].

Extrahepatic metastases following primary HCC treatment are considered signs of widespread microscopic dissemination. For these patients, systematic therapy is considered the golden standard [7]. The molecular-targeted drug sorafenib is expected to prolong the survival of advanced HCC patients following systematic therapy [8]. However, sorafenib has not been shown to provide a survival benefit in patients with extrahepatic metastases [9]. In addition, the optimal treatment of abdominal lymph node metastases in HCC has not been established.

Radiotherapy for metastatic lymph nodes from HCC has been reported as an effective local treatment for palliation [10, 11]. In addition, several studies have reported that local control of metastatic lymph nodes using radiotherapy offers an advantage for improving survival [6, 12–15]. However, in many of these previous studies on the effect of radiotherapy on metastatic lymph nodes, conventional radiotherapy was performed with various therapeutic doses and daily fractions [6, 10]. In addition, radiation complications consistently increased as the radiation dose increased, and gastrointestinal bleeding was a serious complication [12]. The optimal radiotherapy for metastatic lymph nodes from HCC has thus remained unclear.

Stereotactic body radiotherapy (SBRT) has demonstrated efficacy in several patient populations with primary and limited metastatic tumors. SBRT for HCC has demonstrated a high local control rate with acceptable toxicity [16]. However, the literature regarding the efficacy of SBRT for para-aortic lymph node metastases is limited, comprising a few cases of various primary lesions [17–19]. To the best of our knowledge, there is little mention regarding the therapeutic effects of SBRT as a local treatment for regional lymph node metastases in HCC.

The use of SBRT for metastatic lymph nodes in hepatic hilum sites has several limitations due to the poor radiation tolerance of adjacent organs, including the stomach, liver, hepatobiliary system, small intestine, kidneys and spinal cord [20]. Care must be taken to avoid radiation toxicity. In contrast, intensity-modulated radiotherapy (IMRT) has made the delivery of radiation more precise and made the sparing of adjacent organs more efficient [21]. We conducted the present retrospective analysis to evaluate the local control, survival benefit, and toxicity of SBRT delivered using IMRT to oligometastatic regional lymph node metastases in HCC patients.

## MATERIALS AND METHODS

### Patients

We retrospectively analyzed consecutive HCC patients with symptomatic regional lymph node metastases diagnosed and treated with SBRT at our institution between October 2014 and December 2017. This study was performed with the approval of the local Medical Ethics Committee, and written informed consent was obtained from all patients.

The diagnosis of HCC was confirmed by histological examination in all the patients, and the HCCs were treated with TACE and/or RFA. The diagnosis of lymph node metastases from HCC was based on the enhancement pattern observed on contrast-enhanced computed tomography (CT) or MRI studies. The sites of lymph node metastases were the regional lymph node groups composed of the (i) hepatic pedicle, (ii) the retropancreatic space and (iii) the common hepatic artery.

Patients with the following criteria received medical attention for SBRT: (i) symptomatic due to metastatic nodes, (ii) a performance status (PS) of 0–2, (iii) a Child–Pugh Class of A or B, (iv) a controlled intrahepatic tumor without suggestive radiological findings of intrahepatic tumor progression at least before 2 months, (v) a maximum axial diameter of metastatic node < 5 cm and a number of lymph node metastases of ≤ 5, (vi) an absence of direct invasion to adjacent organs such as gastrointestinal organs or main blood vessels and (vii) an absence of distant metastases.

### SBRT and IMRT treatment

Each patient was treated in the supine position with the arms placed above the head. The patient was immobilized by means of a vacuum cushion combined with abdominal pressure corsets to reduce the amplitude of organ motion caused by breathing. In addition, to reduce inter-fractional positioning problems, fasting was required at least 5 h before the treatment session to avoid the hyperperistalsis of stomach for all patients.

Contrast-enhanced 4D computed tomography (4DCT) scanning was carried out during quiet breathing with a 2.5-mm slice thickness on simulation CT scans (GE Medical Systems, Milwaukee, WI, USA). Following 4DCT scanning, images were sorted into 10 phases based on the temporal correlation between the surface motion and the data acquisition on an advanced workstation 4.2 (GE Medical Systems). Contouring and treatment planning were performed using a 3D radiotherapy planning system (Eclipse; Varian Medical Systems, Palo Alto, CA). Gross tumor volumes (GTVs) and clinical target volumes (CTVs) were manually contoured on all 10 phases on the 4DCT scan. The GTV represented the lymph node metastatic lesion visualized on the CT images, and the CTV was defined as the GTV plus an isotropic margin of 3 mm. The internal target volume (ITV) was defined as the combined volume of CTVs in multiple 4DCT phases. The final planning target volume (PTV) was obtained by an additional 3-mm uniform margin expansion of the ITV. Organs at risk (OARs), including the liver, common biliary tract, stomach, duodenum, small intestine, kidneys and spinal cord were contoured. The total liver volume was defined as the total liver volume minus the PTV. Duodenum, small bowel and stomach were contoured until 1 cm above and below the PTV.

All patients were treated by IMRT with 10-MV FF photon beam generated by a Varian linear accelerator (Clinac iX; Varian Medical Systems, Palo Alto, CA). The IMRT plans were designed according to the dynamic sliding window method with fixed gantry beams. Seven to nine co-planar beams with a fixed-jaw setting were applied. The dose calculations and optimizations were performed using the anisotropic analytical algorithm. For the solitary lesions, the dose prescription was set to 45 Gy for the PTV in 6 daily fractions of 7.5 Gy according to the previous study reported by Bignardi *et al* . [22]. For multiple lesions, owing to the prescribed dose having to be downscaled to keep within the dose tolerance of nearby OARs, the dose prescription was set to 49.5 Gy for the PTV in 9 daily 5.5 Gy fractions. The biologically effective doses (BEDs) were 78.8 and 76.7 Gy_10_ (α/β = 10 Gy), respectively. We calculated the dose volume histograms (DVHs) for the PTV, ITV and OARs. For the PTV and ITV, the plan was optimized aiming for *V*_95%_ and *D*_95%_ values > 90%, and *V*_95%_ and *D*_95%_ values > 95%, respectively. For the OARs, the plan needed to meet the following objectives [22, 23]: a V_15Gy_ <100% for the total liver, a *D*_max_ <40 Gy for the common bile duct, a *V*_36Gy_ <1% for the duodenum, a *V*_36Gy_ <3% for the stomach and small intestine, a *V*_15Gy_ <35% for both kidneys and a *D*_max_ <18 Gy for the spinal cord.

For each treatment fraction, the patients were initially set up using the treatment room laser system based on visible marks on the patient’s skin. Then, 2D-orthogonal kilovoltage images were acquired using the on-board imager (OBI, Varian Medical Systems, Palo Alto, CA). The digital reconstructed radiographs (DRR) generated from the planning CT data were used as the reference images. The automatic 2D-matching procedure according to the bony structures between the 2D-orthogonal images and reference DRR images was performed to reposition the patient’s set-up. To verify the accuracy of repositioning, cone-beam CT (CBCT) images were acquired and matched to the planning CT based on 3D anatomic information. When a soft tissue match on the target lesion was unfeasible due to poor image contrast and artifacts on CBCT, matching was performed on the vertebral spine as well as on identified soft tissue structures (e.g. main blood vessels) on the level of the lesion. The CBCT and planning CT were registered to each other by viewing three orthogonal planes using the OBI software in the manual overlay mode. Isocenter shifts in the three orthogonal directions, i.e. the medial–lateral (MR), anterior–posterior (AP) and cranio–caudal (CC) directions, which were determined, applied and defined as the patients positioning error. In cases where the shift was ≤ 3 mm in all three directions, the patients were treated in the corrected position. If the shift was > 3 mm in any direction, an additional CBCT was acquired for verification of the localization after correcting the positioning error. The composite pretreatment verification value was created using a 3D vector calculated by the formula of (MR^2^ + AP^2^ + CC^2^) ^1/2^.

### Treatment response and toxicity

The evaluation of treatment response and follow-up were performed using laboratory assessments and CT and/or MRI imaging. At 8–12 weeks following the completion of the SBRT, patients were monitored using enhanced CT or MRI of the abdomen at the first follow-up visit, and the treatment response was assessed using the Response Evaluation Criteria in Solid Tumors (RECIST). The patients were then followed by CT and/or MRI every 3–6 months. For patients who showed an elevated serum alpha-fetoprotein (AFP) level before SBRT, AFP was assayed at 8–12 weeks following SBRT completion in the first follow-up visit, and AFP changes were assessed in comparison with the levels observed before the treatment. AFP was monitored every 3 months.

Local control was evaluated in terms of freedom from local progression (FFLP), defined as no evidence of regrowth or new tumor inside of the treated volume. The survival was evaluated as the patients’ progression-free survival (PFS) and the overall survival (OS). PFS was defined as the time to regrowth or new tumor in regional nodal area inside or outside of the treated volume, HCC progression, or distant metastasis. The OS period was defined as the time to death due to any cause or the last follow-up visit. The follow-up period was designated as the total time of follow-up starting at SBRT completion and ending at either local recurrence or the last patient contact without local recurrence. FFLP, PFS and OS curves were generated using the Kaplan–Meier method. To evaluate the prognostic factors that had an influence on survival, a log-rank test was used for the univariate analysis and Cox’s proportional hazard model was used for the multivariate analysis. Values of *P* < 0.05 were considered statistically significant. All statistical analyses were carried out with SAS software package version 9.2 (SAS Institute, Cary, NC, USA).

Toxicity from SBRT was graded according to the Common Terminology Criteria for Adverse Events (CTCAE), version 4.0.

## RESULTS

### Patient characteristics

Between October 2014 and December 2017, 15 patients with HCC underwent SBRT delivered using IMRT to 24 regional lymph node metastases. The patients’ mean age was 72.7 years (range 64–82 years). Child–Pugh classification was A in 5 patients and B in the other 10 patients. Intrahepatic tumors were radiologically controlled in all patients. Ten patients had a solitary metastatic node and the other 5 patients had multiple metastatic nodes. The median size of the metastases in the lymph nodes was 2.56 cm. The clinical symptoms presented by the patients before SBRT were abdominal or back pain in all patients and duodenal obstruction in one patient. The median interval between the diagnosis of HCC and the diagnosis of lymph node metastases was 28.5 months (range 14–57 months). Of 15 patients, 3 patients received sorafenib or chemotherapy after SBRT treatment. The patient characteristics are summarized in Table 1.

**Table 1:** Patient characteristics

Characteristic	Value
	No. of patients (*n* = 15)
Age: median, years	72.7
Gender: male/female	6/9
Performance score: 0/1/2	4/7/4
Hepatitis: none/B/C	1/5/9
Child–Pugh classification: A/B	5/10
Intrahepatic tumor number: solitary/multiple	2/13
Number of lymph nodes: solitary/multiple	10/5
Post-SBRT treatment	1
Sorafenib	2
Chemotherapy (5-flurouracil plus cisplatin)	No. of lymph nodes (*n* = 24)
Maximum diameter of lymph node	
≦2 cm	5
2–4 cm	16
≧4 cm	3
Lymph node location	
Hepatic pedicle	12
Retropancreatic space	8
Common hepatic artery	4

### Dose distributions and setup accuracy

Table 2 summarizes the dosimetric characteristics of the treatment plans derived from analysis of the DVHs. The coverage requirement on PTV and ITV was on average respected. The planning objectives for OARs were in general respected as well.

**Table 2:** Summary of the DVH analysis for the treatment plans of all patients

Factor	Volume (cm^3^)	Dose–volume objective	Mean ± SD	Range
PTV	99.6 ± 45.2	*V*_95_ > 90%	92.2 ± 2.9 (%)	89.8–97.1 (%)
		*D*_95_ > 90%	93.9 ± 2.6 (%)	91.9–97.6(%)
ITV	54.9 ± 33.1	*V*_95_ > 95%	99.7 ± 0.5 (%)	99.0–100 (%)
		*D*_95_ > 95%	98.8 ± 2.0 (%)	96.8–100 (%)
Liver	1143.5 ± 522.0	*V*_15Gy_ < 100 (%)	17.9 ± 11.8 (%)	7.3–33.9 (%)
Common bile duct	5.3 ± 3.0	*D*_max_ < 40 Gy	33.5 ± 10.6 (Gy)	15.8–40.7 (Gy)
Duodenum	75.6 ± 58.7	*V*_36Gy_ < 1%	0.1 ± 0.2 (%)	0–0.3 (%)
Stomach	322.4 ± 185.8	*V*_36Gy_ < 3%	2.0 ± 1.4 (%)	0–3.3 (%)
Small bowel	43.6 ± 26.0	*V*_36Gy_ < 3%	0.9 ± 0.9 (%)	0–1.8 (%)
Left kidney	150.5 ± 35.9	*V*_15Gy_ < 35%	13.2 ± 10.2 (%)	0–25.4 (%)
Right kidney	140.2 ± 39.1	*V*_15Gy_ < 35%	9.2 ± 7.9 (%)	0–20.3 (%)
Spine	38.5 ± 8.7	*D*_max_ < 18Gy	14.8 ± 3.2 (Gy)	9.9–17.9 (Gy)

A total of 105 CBCT scans across 15 patients were performed for pretreatment verification. The mean and standard deviation of the registered shifts for the patients positioning error was 0.5 ± 0.3 , 0.2 ± 0.4 and 0.1 ± 0.3 mm in the ML, AP and CC dimensions, respectively. The mean 3D vector was 0.7 ± 0.2 mm. The patients positioning error measured on the pretreatment CBCT was sufficiently small [25]. There was no patient who was required an additional CBCT scan for re-setup.

### Response and survival

**Fig. 1.**
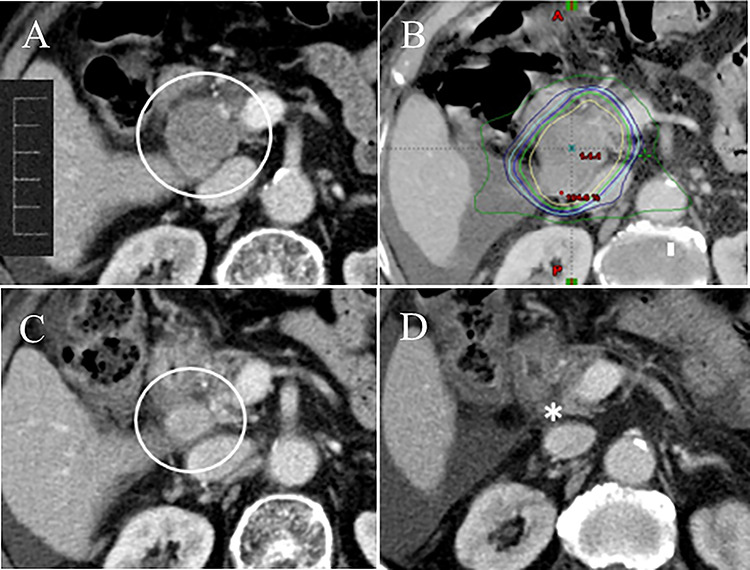
A 70-year-old female with HCC treated with TACE. (**A**) Abdominal CT scan showed an enlarged lymph node metastasis in the retropanceratic space with duodenal obstruction (circle). (**B**) Abdominal CT with dose distribution curves of SBRT delivered with IMRT. The innermost line was the 100% isodose line (yellow line). SBRT was administered with 45 Gy in 6 fractions (BED, 78.8 Gy_10_). (**C**) Abdominal CT at 2 months following the completion of SBRT showed a reduction of the metastatic node size (circle), and the RECIST response rate was partial response (PR). The duodenal obstruction was relieved. (**D**) The metastatic node disappeared on routine follow-up CT at 9 months following the completion of SBRT (asterisk).

Treatment was completed in all 15 patients, and 24 lymph node lesions were treated using SBRT. Among the 15 patients, the RECIST response rate was as follows: (i) complete response (CR) in 10 patients, and (ii) partial response (PR) in the other 5 patients (Fig. 1). The clinical symptoms before SBRT were completely relieved within 12 weeks following the completion of SBRT. Regarding the patients with elevated baseline AFP levels (median: 818.1 μg/L, range: 54.9–2047.0 μg/L), all patients had a reduction in AFP levels after SBRT (median: 201.0 μg/L, range: 21.6–602.2 μg/L).

**Fig. 2.**
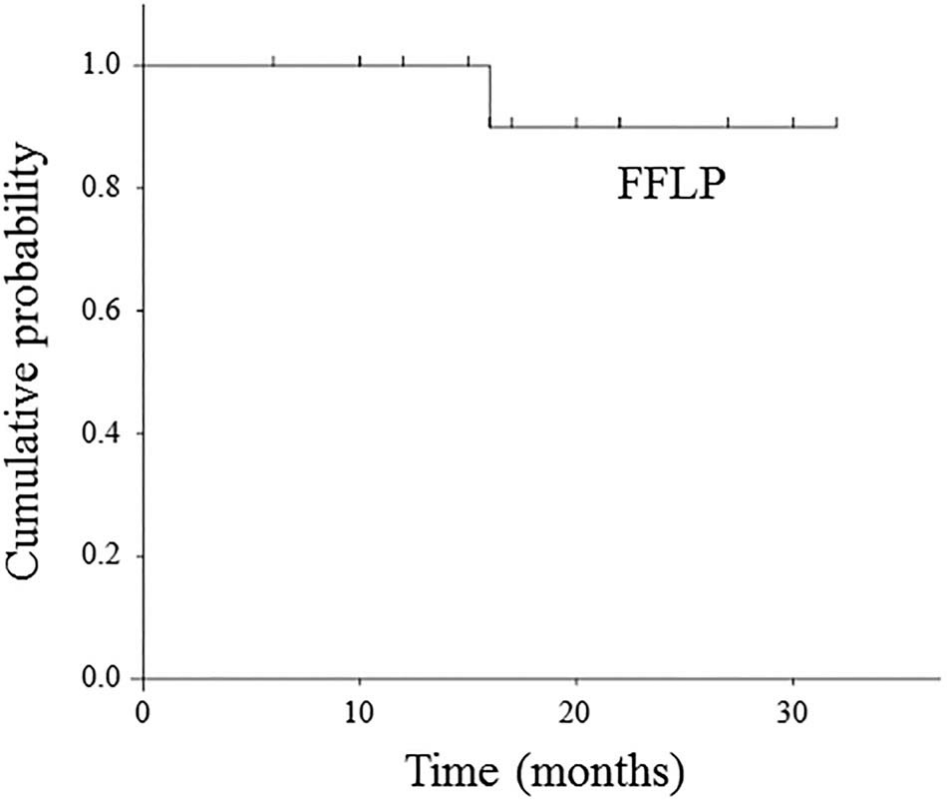
Kaplan–Meier curve of freedom from local progression.

**Fig. 3.**
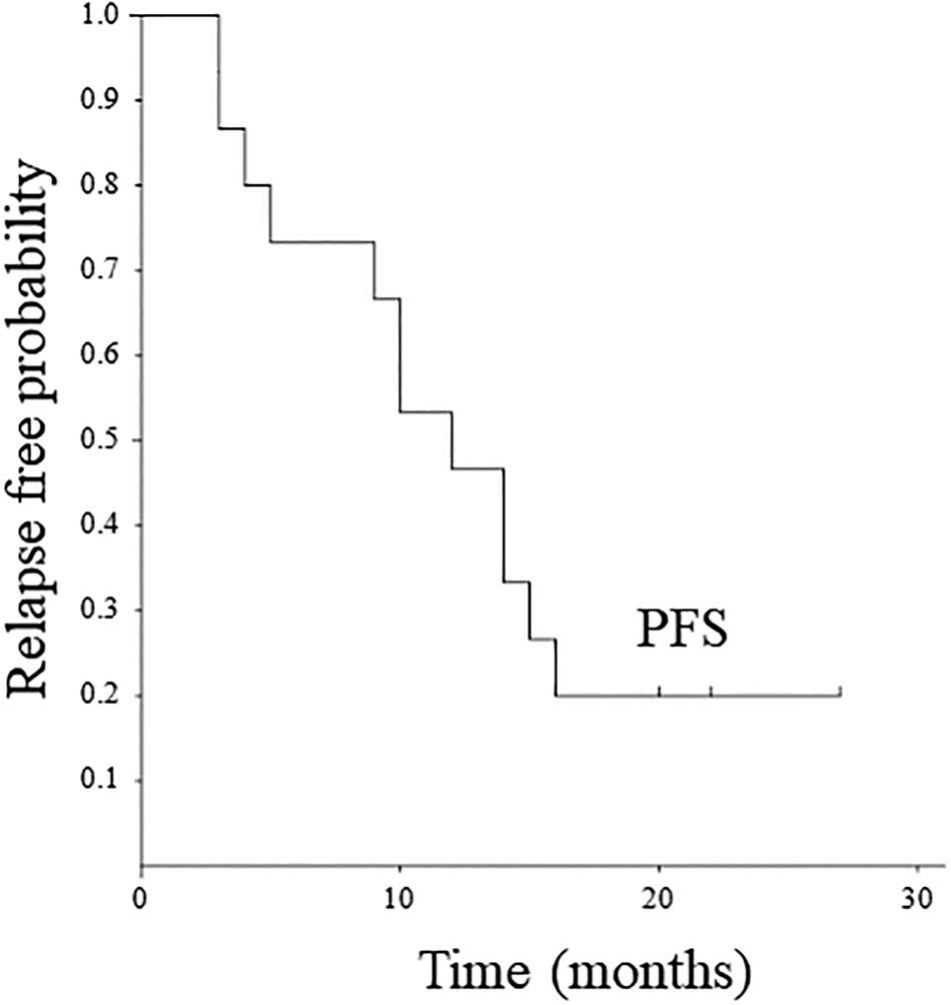
Kaplan–Meier curve of progression-free survival.

**Fig. 4.**
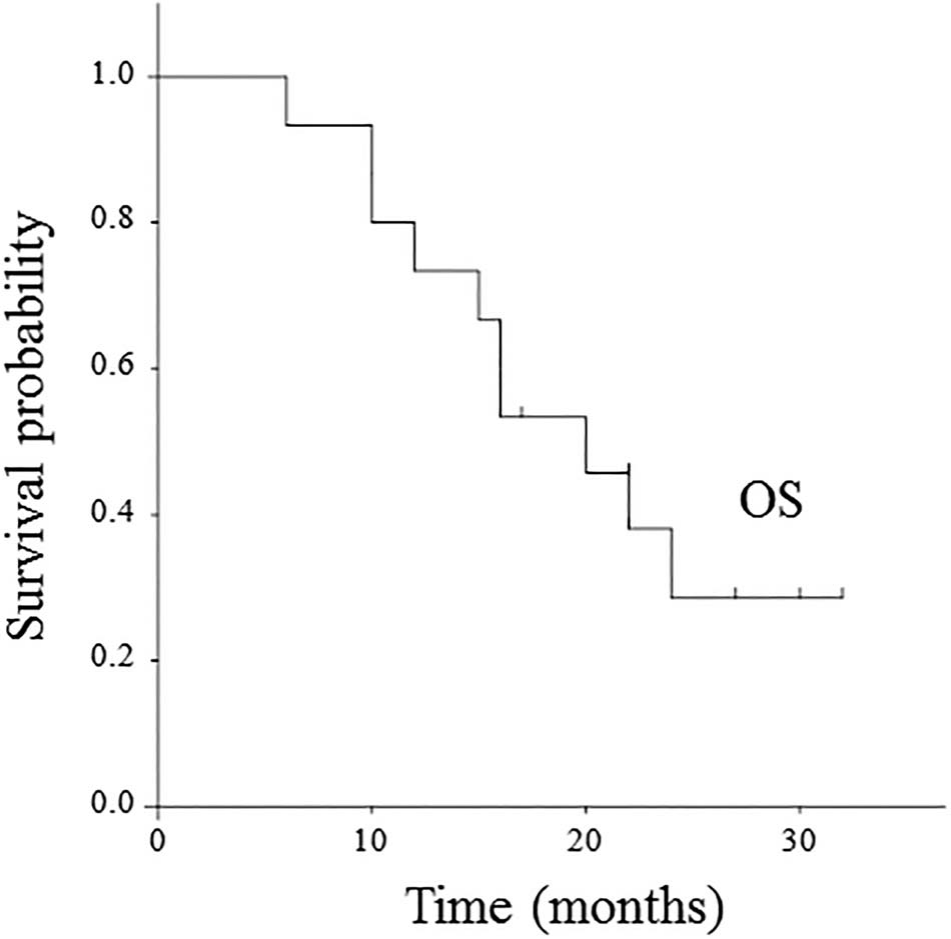
Kaplan–Meier curve of overall survival.

The median follow-up period was 18.1 months (range: 6–32). One patient had a local progression inside of the treated volume at 16 months, and also showed concomitant HCC progression. Additionally, 11 patients showed regional node progression, HCC progression, or distant metastasis during the follow-up period: (i) 8 patients had only HCC progression, (ii) 1 patient had regional node progression outside of the treated volume and concomitant HCC progression, and (iii) 2 patients had HCC progression and concomitant distant metastasis in the lung. Consequently, 12 patients developed disease progression during the follow-up period. Time to progression ranged from 3 to 16 months. In the Kaplan–Meier analysis, the 1-year and 2-year FFLP rates were 100 and 90.0 ± 9.5%, respectively (Fig. 2). The 1-year PFS rate was 46.7 ± 12.9% (Fig. 3). At the time of data analysis, 5 of the 15 patients were alive. The median survival time was 18.6 months. The 1-year and 2-year OS rates were 73.3 ± 11 .4 and 28.6 ± 12.7%, respectively (Fig. 4).

As summarized in Table 3, only Child–Pugh classification was associated with OS on univariate analysis (*P* = 0.04), however, other parameters were not significant factors in PFS and OS. In addition, on multivariate analysis, there were no significant factors affecting PFS and OS.

**Table 3:** Univariate analysis of prognostic factors associated with.progression-free survival and overall survival.

Factor (*n*)	Progression-free survival	Over all survival
	*P* value	*P* value
Age, years		
≦70 (6)	NA	NA
>70 (9)		
Gender		
Male (6)	NA	NA
Female (9)		
Performance score		
0, 1 (11)	NA	NA
2 (4)		
Child–Pugh classification		
A (5)	NA	0.04
B (10)		
Number of lymph nodes		
Solitary (10)	NA	NA
Multiple (5)		
Maximum diameter of lymph node		
≧3.5 cm (14)	NA	NA
<3.5 cm (10)		
Regional lymph node location		
Hepatic pedicle only (7)	NA	NA
Others (17)		
Response to SBRT		
CR (10)	NA	NA
PR (5)		
Chemotherapy after SBRT		
Yes (3)	NA	NA
No (12)		

### Toxicity

There was no patient with toxicity of Grade 3 or higher during the follow-up period. Three patients had acute hematologic toxicity (Grade 1), including a decrease of neutrophils and/or platelets. Six patients experienced Grade 1 or 2 sub-acute toxicity; 2 patients had gastritis (Grade 1), only 1 patient had a duodenal ulcer (Grade 2) that was treated with an antacid drug, and 3 patients had liver enzyme elevation (Grade 1). Late toxicity was not observed during the follow-up period.

## DISCUSSION

It has been reported that the presence of lymph node metastasis from HCC should be considered as an indicator of the aggressiveness of HCC as a whole, rather than an independent prognostic determinant [10, 11]. However, the prognosis is better among patients with HCC who receive some treatment for their lymph node metastasis compared to those who are left untreated, and radiotherapy has been an effective local treatment [6, 12–15]. In addition, several studies have reported significant prognostic factors for radiotherapy to lymph node metastasis from HCC, such as the patient’s responsiveness to radiotherapy, the Child–Pugh classification, the HCC status, the presence of distant metastasis, the location and number of metastatic nodes and the presence of lymph node related symptoms [12–15]. Radiotherapy for lymph node metastasis should thus be considered in patients with controlled HCC and lymph node-related symptoms, and without poor hepatic function or synchronous distant metastasis.

Although the optimal radiation dose and daily fraction to metastatic nodes in HCC patients have been unclear, several research groups using conventional radiotherapy have suggested that a radiation dose of 45–50 Gy_10_ with a daily dose of 2–3 Gy is required to achieve a major response from lymph node metastases in HCC [6, 10–15]. Park *et al*. [12] have reported a 79.5% response rate using a total dose of 39–58.5 Gy_10_ with a daily dose of 1.8–3 Gy. Kim *et al*. [15] have reported that the response rate increases in parallel with the radiation dose, being 58.3, 62.9 and 76.1% in patients receiving radiation doses of ≤ 40, 41–50 and > 50 Gy_10_, respectively. In addition, Wee *et al*. [14] have reported that a radiation dose of > 60 Gy_10_ is necessary for achieving a favorable radiological response to metastatic nodes from HCC.

On the other hand, regarding the treatment response to SBRT for abdominal lymph node metastases, Yeung *et al*. [19] have reported a 1-year local control rate of 94% in oligometastatic lymph nodes from several primary lesions treated with SBRT using a total dose of 54.8–105 Gy_10_ in 4–10 fractions. In addition, Choi *et al*. [17] have observed a 4-year actuarial local control value of 67.4% in para-aortic lymph node metastases from cervical and corpus cancer treated with 69.3–112.5 Gy_10_ in 3 fractions of SBRT. Although there are few reports regarding SBRT to regional lymph node metastases of HCC, in the present study, using SBRT with a total dose of 76.7–78.8 Gy_10_ in 6–9 fractions the objective response (CR + PR) rate of RECIST was 100%, and the 1-year local control rate of 100% was achieved. Additionally, the patients’ clinical symptoms before SBRT were completely relieved. The results of our analyses, thus, suggest that SBRT can provide an excellent local control in metastatic nodes from HCC, better than conventional radiotherapy.

Although the survival results following radiotherapy to metastatic nodes in HCC patients may be influenced by the HCC status, hepatic function and distant metastasis, several studies have reported that the median survival time and the 1-year survival rates range from 7–13 months and 31–53%, respectively [6, 10–12]. In a recent study of risk factors predicting overall survival following radiotherapy to metastatic nodes in HCC patients by Kim *et al*. [15], the Child–Pugh classification, the intrahepatic tumor status, the presence of distant metastasis, the number and location of metastatic nodes, the serum level of AFP and the response to radiotherapy were significant risk factors for OS. In this study, the median OS according to numbers of risk factors were 2.9, 5.5, 10.3, 13.6 and 27.8 months in patients with ≥4, 3, 2, 1 and 0 risk factors, respectively.

Similarly, Wee *et al*. [14] have reported that patients with no risk factors can achieve a long-term median survival of 18 months following radiotherapy, whereas patients with three or more risk factors demonstrate a very poor median survival of 3 months. In the present study, although most of the patients had a few risk factors (i.e. Child–Pugh class B, multiple lymph node metastases, and lymph node-related symptoms), the median survival time and 1-year OS were 18.6 months and 73.3 ± 11.4%, respectively. An improvement of the prognosis of HCC patients with metastatic nodes may therefore be anticipated when using SBRT in metastatic nodes. However, regarding the analysis of prognostic factors, the parameters such as number of lymph nodes, maximum diameter of lymph node, regional lymph node location and response to SBRT were not significant factors affecting survival. Therefore, further studies are necessary to identify the survival benefit and the prognostic factors of SBRT to metastatic nodes in HCC patients.

In previous studies of radiotherapy to abdominal lymph node metastases in HCC patients, gastrointestinal bleeding was reported as one of the most serious forms of toxicity [6, 10, 12]. According to Zeng *et al*. [6], the incidence of gastrointestinal bleeding exceeded 40% in patients treated with > 67.2 Gy_10_ in daily 2-Gy fractions. In our present series, there was no patient with Grade 3 or higher toxicity; there was a single patient with a Grade 2 duodenal ulcer. High doses of radiation to the centrally oriented biliary system and vasculature have the potential for severe toxicity.

There have been few studies evaluating the hepatobiliary toxicity associated with SBRT treating liver tumors adjacent to the central biliary system [23, 24]. Eriguchi *et al*. [23] have reported that SBRT with a dose of 72 Gy_10_ in 5 fractions was safe and feasible with minimal hepatobiliary toxicity when the biliary tact irradiated with > 36 Gy_10_ was < 7 mm. In addition, Osmundson *et al*. [24] have suggested *V*_BED10_ 72 Gy < 21 cm^3^ and volume receiving above BED10 66 Gy < 24 cm^3^ as potential dose constraints for the central biliary system in SBRT treating liver tumors. Only 3 of our patients had a liver enzyme elevation of Grade 1 following SBRT, despite undergoing radiotherapy to lymph node metastases adjacent to the central biliary system and portal vein. The incidence and level of toxicity of SBRT were thus minimal in our patient cohort. This result might be explained by the fact that the lymph node metastases in our patients were treated with SBRT delivered with IMRT to avoid radiation toxicity.

Our study has several limitations. The results are from a single-institution retrospective analysis with a small number of patients and a short follow-up. This has limited our ability to report and capture the results of patients who met our eligibility criteria. However, this study is a preliminary report evaluating the local control, survival benefit and toxicity of SBRT delivered with IMRT to regional lymph nodes metastases in HCC patients. In addition, in this study, patients with symptoms such as abdominal or back pain due to metastatic nodes were eligible for SBRT, however, asymptomatic patients might also be eligible for SBRT. A larger retrospective cohort and prospective clinical studies are necessary to determine the clinical benefits of SBRT for eliminating lymph node metastasis as well as to identify the subset(s) of patients who benefit the most from SBRT.

In HCC patients with symptomatic regional lymph node metastasis, SBRT delivered with IMRT to the lymph node metastases can provide excellent local control with minimal toxicity. SBRT may better improve these patients’ survival compared to conventional radiotherapy.

## CONFLICT OF INTEREST

None declared.
